# Dendritic Inhibition Effects in Memory Retrieval of a Neuromorphic Microcircuit Model of the Rat Hippocampus

**DOI:** 10.3390/brainsci15111219

**Published:** 2025-11-13

**Authors:** Nikolaos Andreakos, Vassilis Cutsuridis

**Affiliations:** 1 School of Computer Science, University of Lincoln, Lincoln LN6 7TS, UK; 2 School of Engineering, Computing and Mathematics, University of Plymouth, Drake Circus, Plymouth PL4 8AA, UK

**Keywords:** dendrites, CA1, memory recall, engram cell, theta rhythm, inhibition

## Abstract

**Background:** Studies have shown that input comparison in the hippocampus between the Schaffer collateral (SC) input in apical dendrites and the perforant path (PP) input in the apical tufts dramatically changes the activity of pyramidal cells (PCs). Equally, dendritic inhibition was shown to control PC activity by minimizing the depolarizing signals in their dendritic trees, controlling the synaptic integration time window, and ensuring temporal firing precision. **Objectives:** We computationally investigated the diverse roles of inhibitory synapses on the PC dendritic arbors of a CA1 microcircuit model in mnemonic retrieval during the co-occurrence of SC and PP inputs. **Results:** Our study showed inhibition in the apical PC dendrites mediated thresholding of firing during memory retrieval by restricting the depolarizing signals in the dendrites of non-engram cells, thus preventing them from firing, and ensuring perfect memory retrieval (only engram cells fire). On the other hand, inhibition in the apical dendritic tuft removed interference from spurious EC during recall. When EC drove only the engram cells of the SC input cue, recall was perfect under all conditions. Removal of apical tuft inhibition had no effect on recall quality. When EC drove 40% of engram cells and 60% of non-engram cells of the SC input cue, recall was disrupted, and this disruption was worse when the apical tuft inhibition was removed. When EC drove only the non-engram cells of the cue, then recall was perfect again but only when the population of engram cells was small. Removal of the apical tuft inhibition disrupted recall performance when the population of engram cells was large. **Conclusions:** Our study deciphers the diverse roles of dendritic inhibition in mnemonic processing in the CA1 microcircuit of the rat hippocampus.

## 1. Introduction

**Figure 1:**
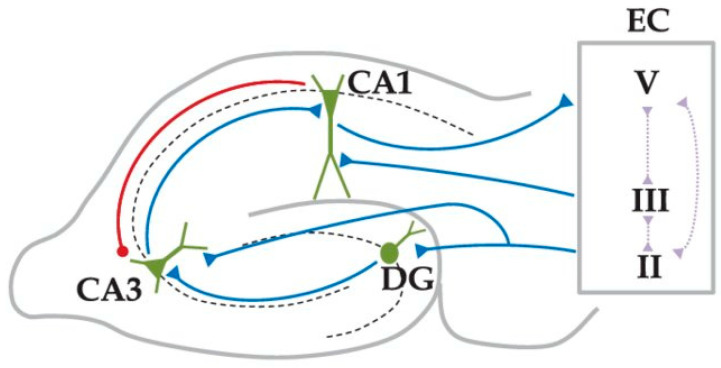
Schematic representation of the hippocampal circuit (adapted with permission from [10], Figure 1, © AIP Publishing). EC-L2 input excites granule cells (GC) in the dentate gyrus (DG) and distal dendrites of CA3 PCs. DG-GCs excite the proximal dendrites of CA3 PCs. CA3 excites the proximal dendrites of CA1 PCs via the SC path, while the EC-L3 excites the distal dendrites of CA1 PCs via PP. CA1 PCs then drive EC L5 cells.

Theoretical studies [1] have hypothesized that in the hippocampus, context-dependent memory sequence retrieval and novelty detection are mediated by input comparison of the PP and SC inputs. In CA1, PP provides direct input from layer 3 of the entorhinal cortex to the apical dendritic tuft of the CA1 PCs, whereas SC provides input to their basal and apical dendrites via the trisynaptic loop (see Figure 1) [2,3]. Experimental studies have shown that interaction between PP and SC inputs results in changes in synaptic plasticity and inhibitory input, and to an amplified propagation of distal dendritic Na+ spikes [4,5,6,7,8,9].

Takahashi and Magee [9] observed that coincident activation of SC and PP inputs generates long-lasting dendritic plateau potentials, which in turn produce an after-depolarization in the soma and axon regions that shifts the firing output mode from regular spiking to burst firing [11,12].

Control of PC firing mode is also possible by the myriads of inhibitory synapses localized in their soma, axon, and dendritic arbors [13]. Dendritic inhibition controls PC activity by restricting the occurrence of depolarizing signals within their dendritic trees, controlling the time window for synaptic integration, and ensuring temporal firing precision at the circuit level [14,15,16].

In our study, we have computationally investigated the diverse roles of inhibitory synapses on the PC dendritic arbors of a CA1 microcircuit model in mnemonic processing during a multiplicative interaction of SC and PP inputs. Our CA1 microcircuit model consisted of a population of PCs and two types of dendritic inhibitory neurons: bistratified cells (BSC) targeting the apical PC dendrites and oriens lacunosum-moleculare (OLM) cells targeting the apical PC dendritic tufts. Our study showed that BSC inhibition in the apical PC dendrites mediates thresholding of firing during memory retrieval by restricting the depolarizing signals in the dendrites of non-engram cells, thus preventing them from firing, and ensuring perfect memory retrieval (only engram cells fire). On the other hand, OLM inhibition mediates interference removal from spurious EC activity in the apical dendritic tuft during recall. When EC drives only the engram cells in the SC cue, then recall is perfect under all tested conditions (stored patterns, interference, engram cells). Removal of apical tuft inhibition (OLM) has no effect on recall quality. When EC drives 40% of the engram cells and 60% of non-engram cells in the SC cue, then recall is disrupted, and this disruption is worse when the apical tuft inhibition is removed. When EC drives only the non-engram cells of the SC cue, then recall is perfect again but only when the population of engram cells is small. Removal of the apical tuft inhibition disrupts recall performance when the population of engram cells is large.

The remainder of this article is organized as follows: Section 2 summarizes experimental evidence tying theta rhythm to memory formation (encoding and retrieval) and how CA1 and medial septal (MS) cells (excitatory and inhibitory) fire with respect to the theta rhythm. Section 3 briefly presents the CA1 circuit model, its inputs, how its recall performance is calculated, and the simulation environment and data analysis tools used. Section 4 presents comprehensive experiments, including recall performance evaluation, and comparison and contrast of six models’ performance with and without BSC and OLM inhibition. Section 5 discusses our model’s predictions and offers alternatives/extensions worth of further investigation under the prism of theoretical memory neuroscience. Section 6 summarizes the work.

## 2. Background

Theta rhythm (4–10 Hz) [17,18,19] has been implicated in memory formation [20,21], with memory encoding and retrieval occurring at distinct phases of a theta cycle [22]. Disruption of the theta rhythm has been shown to result in behavioral deficits in animals [23]. In the hippocampus, theta rhythm is paced by the MS and the diagonal band of Broca in the basal forebrain [23,24]. The hippocampus also has its own internal mechanisms to maintain theta in the absence of an external oscillatory signal [25,26]. Theta oscillators have been recorded in stratum lacunosum moleculare (SLM) in CA1, in stratum moleculare (SM) in dentate gyrus (DG), and stratum pyramidale (SP) in CA1 [27]. The SLM oscillator has been found to oscillate at the opposite phase of the SM and SP oscillators, whereas the SM and SP oscillators oscillate in phase with each other [27].

Excitatory and inhibitory neurons in the hippocampus fire at different phases of the theta rhythm [28,29]. CA1 dendritic inhibitory cells (OLMs and BSCs) and PCs fire at the trough of theta recorded in the CA1 SP, whereas CA1 axonic (axo-axonic cells (AACs)) and somatic (basket cells (BCs)) inhibitory cells fire at the peak of theta recorded in the CA1 SP [28,30,31]. CA1 inhibition sculpts the activities of PCs, allowing them to fire at particular temporal windows and phases of theta [28,32]. In addition to hippocampal cells, MS cell activities are theta-modulated [33,34,35]. Inhibitory MS neurons are of two types, exhibiting highly regular bursting activity, with one type coupled to the trough of hippocampal theta and the other coupled to the peak of hippocampal theta [33].

## 3. Materials and Methods

### 3.1. The Model

**Figure 2:**
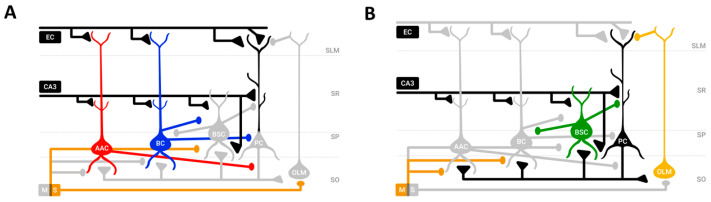
CA1 encoding (**A**) and retrieval (**B**) microcircuits showing cell types and their connectivity. Colored-filled circles, active inhibition; gray-filled circles, inactive inhibition; black-filled reverse triangles, active excitation; gray-filled reverse triangles, inactive excitation; EC, entorhinal cortex input; CA3, CA3 Schaffer collateral input; AAC, axo-axonic cell; BC, basket cell; BSC, bistratified cell; OLM, oriens lacunosum-moleculare cell; MS, medial septum; SLM, stratum lacunosum-moleculare; SR, stratum radiatum; SP, stratum pyramidale; SO, stratum oriens (adapted with permission from [36], Figure 1, © Springer Nature).

Our model was the Andreakos et al. [36] neurobiologically plausible microcircuit model of region CA1 of the rat hippocampus [37,38,39]. No changes or extensions to [36] were made in our study. The model consisted of two microcircuits: an encoding microcircuit (see Figure 2A) active during the memory encoding phase (peak of an externally imposed theta oscillation), and the retrieval microcircuit (see Figure 2B) active during the memory retrieval phase (trough of the externally imposed theta oscillation). The model consisted of 100 Hodgkin–Huxley-based excitatory cells (PCs) and 4 types of inhibitory neurons (AACs, BCs, BSC, OLM cells) tuned to fire at different phases of an externally imposed theta oscillation by two opponent firing populations of MS cells (MS_1_ and MS_2_) targeting only the inhibitory cells in the network. The inhibitory cells in the network, in turn, sculpt the activities of PCs to fire at specific time windows (see Section 2).

The morphologies of the PCs, AAC, BCs, BSC, and OLMs have been described in detail in [36]. Interested readers should refer to the [36] paper for details. Details of the dimensions of cells’ somatic, axonic, and dendritic compartments and distributions of passive and active conductance along these compartments can be found in Supplementary Tables S1–S6 of the Supplementary Online Material (SOM) of [36]. The complete mathematical formalism of the model can be found in the SOM of [36]. Excitatory (AMPA and NMDA) and inhibitory (GABA-A and GABA-B) synapses were present in the network. GABA-A and GABA-B were present in soma, axon, and dendrites of all cells. AMPA were present only in cell dendrites, and NMDA were present only in PC dendrites. The parameters for the synaptic waveforms and synaptic conductance can be found in Supplementary Tables S7 and S8 of [36], respectively. Voltage traces of all cells in response to depolarizing and hyperpolarizing current injections can be seen in Supplementary Figures S2, S4 and S6 of [36].

**Figure 3:**
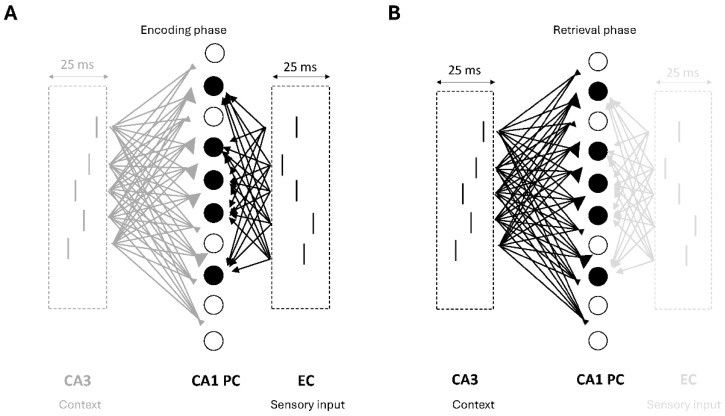
Model inputs to the CA1 microcircuit. Solid black circles: engram cells. Hollow circles: non-engram cells (spurious cells). EC, entorhinal cortex input (spikes) with a 25 ms jitter. CA3, Schaffer collateral input (spikes) with a 25 ms jitter. (**A**) During the encoding, a strong EC input (black spikes) is associated with a weak CA3 input (heavy gray spikes). The association is accomplished via an STDP learning rule in the medial SR dendrites of a selected group of PCs (engram cells). The EC input selectively excites a subset of PCs (engram cells) in the network, whereas the CA3 input non-selectively excites all PCs (engram and spurious cells). The synaptic weights to the engram cells are stronger (large-filled reverse triangles) than the synaptic weights of other cells (small-filled reverse triangles). (**B**) During the retrieval phase in the absence of an EC input (light gray spikes), a strong CA3 input (black spikes) non-selectively excites all PCs (engram and spurious cells). It is expected that only the engram cells (cells with large-filled reverse triangles) will fire, while spurious cells (cells with small-filled reverse triangles) will be silenced due to a non-specific BSC inhibition, which acts as a threshold mechanism.

**Figure 4:**
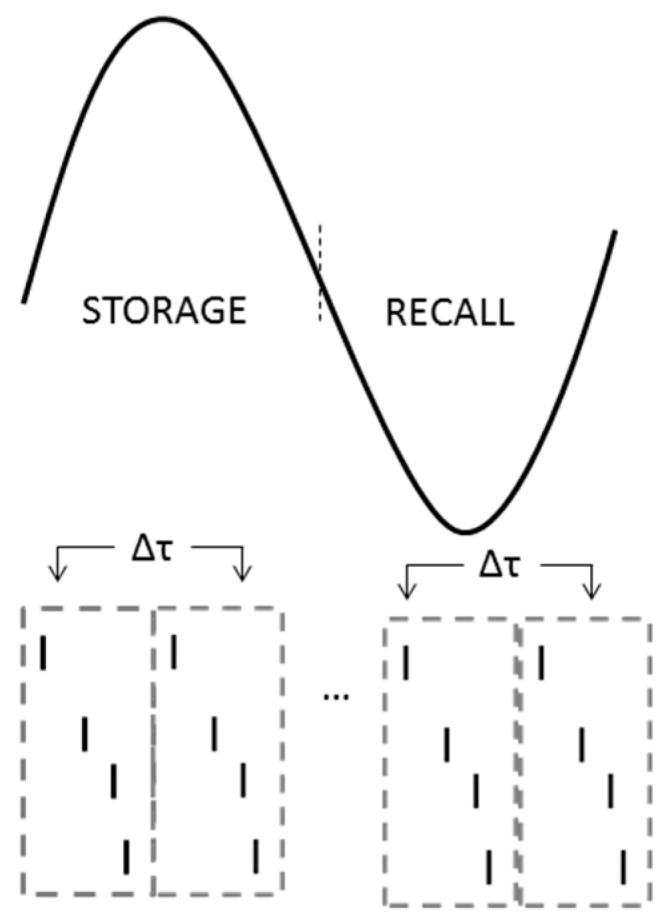
Input presentation in the model. Inputs are presented continuously at gamma frequency throughout the storage and recall cycles of the theta rhythm. Each input window (gray rectangular window) is repeated every Δτ = 25 ms (adapted with permission from [38], Figure 3, © Springer Nature).

The model cells were driven by two excitatory inputs, a sensory input from the entorhinal cortex (EC) via PP and a contextual one from CA3 via SC, both targeting the dendrites of cells in the network and causing them to fire action potentials (spikes). The EC input represented the sensory input (memory pattern) to be learned during encoding and retrieved during recall, whereas the CA3 input represented the contextual information, which was associated with the sensory input during encoding and used as a cue during the retrieval. A presynaptic spike generator with noise was used to generate the firing of S (S = 5, 10, 20, unless otherwise stated) EC spike trains presented at an average 40 Hz gamma frequency. (see Figure 3 and Figure 4). Another presynaptic spike generator with noise was used to generate the firing of M (M = 5, 10, 20, unless otherwise stated) out of N PCs CA3 spike trains at an average 40 Hz gamma frequency (see Figure 3 and Figure 4). Two other presynaptic spike generators were used to generate the bursts of action potentials at a mean frequency of 8 Hz for a half-theta cycle (70 ms), followed by a half-theta cycle of silence of each MS population of 10 septal cells (MS_1_ and MS_2_) modulated at opposite phases of a theta cycle (180° out of phase) [33,40]. Due to 8% noise in the inter-spike intervals (ISIs), the 10 spike trains in each septal population were asynchronous.

### 3.2. Cell Firings During the Encoding and Retrieval Theta Cycles

During the encoding phase (peak of theta) (see Figure 3 and Figure 4 ), a strong excitatory synaptic input from EC drove the distal dendrites of AAC, BCs, and PCs [27], while a weak CA3 input drove the proximal dendrites of AAC, BCs, BSC, and PCs [27]. The maximally active AAC and BCs [28,30,31] inhibited the weakly active BSC and the PCs. The PCs excited the OLM cells. The maximally active MS cells (MS_1_) [33] silenced the BSC and OLM cells. The distal PC dendritic postsynaptic potentials due to the strong EC input and the proximal PC dendritic postsynaptic potentials due to the weak CA3 input provided a strong depolarized potential in the proximal PC dendrites to drive associative learning via spike timing-dependent plasticity (STDP).

During the retrieval phase (trough of theta) (see Figure 3 and Figure 4), AAC and BCs were silent [28,30,31] due to active MS_2_ inhibition [33], whereas PCs, BSC and OLM cells were maximally active [28,30,31] due to a strong CA3 input which excited the proximal dendrites of AAC, BCs, BSC and PCs [27], while a weak EC input had a minuscule effect on the distal dendrites of AAC, BCs and PCs [27]. During this phase, associative learning was paused.

### 3.3. CA1 Model Variations

**Figure 5:**
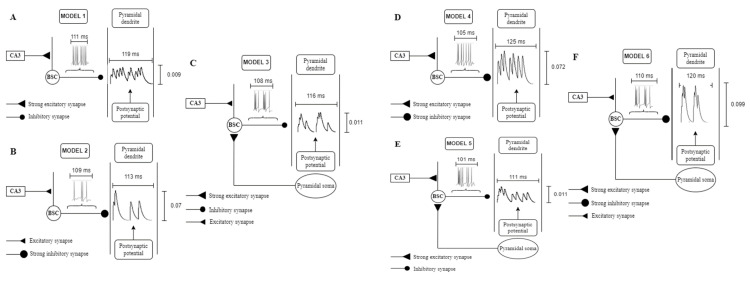
Schematic drawings of all six models of the retrieval microcircuit. (**A**) In ‘Model 1’, a strengthened excitatory synapse in the BSC proximal to the soma dendrite driven by the CA3 input increases the BSC firing response, which in turn generates in the PC dendrite numerous facilitated small amplitude with small duration that never decay to zero summed postsynaptic potentials, thus producing a very strong steady inhibitory environment which filters out spurious activities. (**B**) In ‘Model 2’, a strengthened inhibitory synapse in the PC dendrite driven by BSC produces fewer with larger amplitude and duration postsynaptic potentials. (**C**) In ‘Model 3’, a strengthened excitatory synapse in the BSC basal dendrites driven by the feedback excitatory PC signal increases the BSC firing response, which in turn generates fewer than ‘Model 1’ postsynaptic potentials in the PC dendrite, and hence a less strong, but steady inhibitory environment than ‘Model 1’. (**D**) ‘Model 4’ was the combination of ‘Model 1’ driven by a one-third in strength excitatory CA3 input and ‘Model 2’. In the PC dendrite of ‘Model 4’ more postsynaptic potentials with a larger amplitude and smaller duration than ‘Model 2’ postsynaptic potentials. (**E**) ‘Model 5 was the combination of ‘Model 3’ and ‘Model 1’ driven by a one-third in strength excitatory CA3 input. (**F**) ‘Model 6’ was the combination of ‘Model 2’ and ‘Model 3’ (adapted with permission from [13], Figure 5, © Springer Nature).

Andreakos and colleagues [36] identified 6 different synaptic pathways (models) in the retrieval microcircuit model capable of having an effect on its memory recall performance:

1. Model 1: Scaling of excitatory synapses in the proximal to the soma BSC dendrites. These synapses were excited by feedforward excitatory Schaffer collateral CA3 input (Figure 5A).
2. Model 2: Scaling of inhibitory synapses from the medial to the soma PC dendrites. The synapses are excited by feedforward excitatory Schaffer collateral CA3 input and inhibited by the BSC inhibition (Figure 5B).
3. Model 3: Scaling of excitatory synapses in the BSC basal dendrites. These synapses were excited by PC feedback excitation (Figure 5C).
4. Model 4: Model 1 and Model 2 (Figure 5D).
5. Model 5: Model 1 and Model 3 (Figure 5E).
6. Model 6: Model 2 and Model 3 (Figure 5F).

### 3.4. Memory Patterns

Memory patterns were created using the same method reported in [36]. Briefly, sets of memory patterns of different sizes (1, 5, 10, 20), percent overlap between patterns (40%), and number of engram cells (EngC) (5, 10, 20) coding for a memory pattern were created using an in-house built Python 3.0 script. For example, a 40% overlap between 10 patterns in a set meant that the 0.4 × Μ cells shared by a pattern pair (e.g., 1 and 2) are not shared by any other pattern pair (e.g., 3 and 4, 4 and 5, 5 and 6, …, or 10 and 1). Thus, in a network of 100 PCs where 10 EngCs represent a memory pattern and 10% overlap is between stored patterns, the maximum number of patterns the network’s weight matrix could store is 10.

### 3.5. Weight Matrix

CA3 to CA1 PC connection weights (weight matrix) were generated using the method described in [37]. We assumed that the input (CA3) and output (CA1) patterns were the same, with each pattern consisting of M (M = 5, 10, 20, unless otherwise stated) randomly chosen PCs (engram cells) out of the population of N PCs. If the input PC i and output PC j were both active in the same pattern pair, then the matrix entry (i, j) was set to 1 (w_ij_ = 1); otherwise, the weight entry was set to 0. An NxN weight matrix was created, with some entries being 1 and others being 0. The matrix was applied to our network model by connecting a CA3 input to a CA1 PC with a high AMPA conductance (gAMPA = 1.5 nS) if their connection weight was 1, or with a low conductance (gAMPA = 0.5 nS) if their connection was 0. This approach was supported by experimental evidence favoring two-state synapses [41].

### 3.6. Recall Quality and Mean Recall Quality

Recall quality (*RQ*) was calculated as the normalized dot product metric (distance) between the recalled output pattern, *A*, from the required output pattern, A*:

(1) $$RQ=\frac{A\cdot A^{*}}{\left(\sum_{i=1}^{NA}A_{i}\cdot \sum_{j=1}^{NA}A_{j}^{*}\right)}$$

where *N_A_* was the number of output units. *RQ* took value from 0 (no correlation between output pattern *A* = [1 0 1 0 1 0] and output pattern A* = [0 1 0 1 0 1]) to 1 (output pattern *A* = [1 0 1 0 1 0] and output pattern A* = [1 0 1 0 1 0] were identical). The higher the correlation value, the better the *RQ*.

Mean recall quality (*MRQ*) was calculated as the mean value of all recall qualities estimated from each pattern presentation when a *P* number of patterns were already stored in the network:

(2) $$MRQ=\frac{\sum_{i=1}^{N_{p}}{RQ}_{i}}{N_{p}}$$

where *RQ_i_* was the recall quality of pattern *i* and *N_p_* was the total number of recalled patterns.

### 3.7. Simulation Environment

All simulations were performed with NEURON [42] running on a small network of 4 personal computers with 4 CPUs each under Windows 10. Analysis of data was performed with in-house built MATLAB2021 and Python scripts.

## 4. Results

### 4.1. Pure Recall of Previously Stored Patterns

A set of patterns were stored by generating a weight matrix that prespecifies the CA3 to CA1 PC connection weights (see Section 3.5 for details). Recall of a pattern from a set of P stored patterns was tested by presenting the associated input pattern to the network as a cue in the form of spiking of active CA3 inputs (those belonging to the pattern) distributed within a gamma frequency time window. The entire cue pattern was repeated at gamma frequency (40 Hz; Figure 4). At the same time, 20 EC inputs fired randomly within a 25 ms gamma window and drove the apical tuft dendrites of the inhibitory neurons and the PCs. All inhibitory network cells were switched on and off as we described in Section 3.2. The CA3 input drove the CA1 AAC, BC, BSC, and PCs. The EC input only drove the AAC and BC. To test pure recall by the CA3 SC input cue, the EC input was disconnected from the CA1 PCs. Recall occurred only during the ‘‘recall’’ half-cycle.

**Figure 6:**
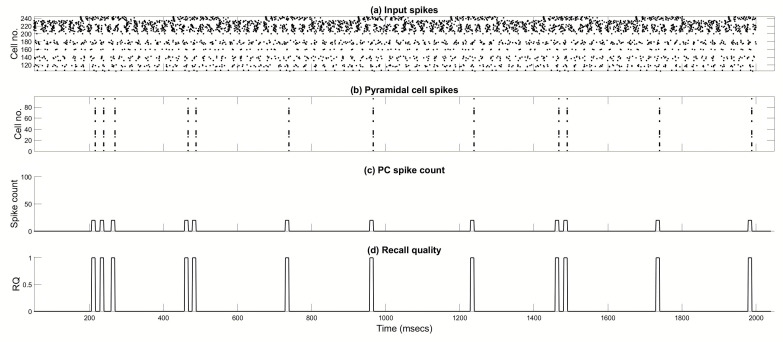
Example of a pattern recall. The CA3 input is cueing the first pattern in a set of five stored patterns. EC input is present to drive the inhibitory interneurons but is disconnected from the CA1 PCs so that recall is purely due to the CA3 input cue. Eight 125 ms recall half-cycles are shown, starting at 50 ms (interspersed with 125 ms storage half-cycles, but STDP is turned off). (**a**) Raster plot showing the MS_1_ (top 10), MS_2_ (next 10), EC (next 20), and CA3 input (bottom 100) spikes. (**b**) Raster plot showing EngC activity. (**c**) PC spike count in a sliding 10 ms bin. (**d**) Recall quality in a sliding 10 ms bin.

**Figure 7:**
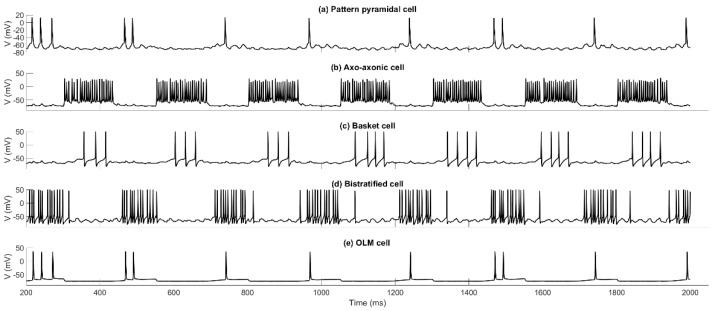
Voltage traces in an engram cell (a PC belonging to the pattern), and each type of inhibitory interneuron with respect to external theta oscillation. Pattern PC fires in phase with BSC and OLM and out of phase with AAC and BC, which fire in phase with each other. Eight 250 ms recall theta-cycles are shown, starting at 200 ms.

Figure 6 shows the recall of the first pattern in a set of five stored patterns. Figure 6a is a raster plot of the MS_1_ (top 10 rows), MS_2_ (next 10 rows), EC (next 20 rows), and CA3 (bottom 100 rows) input spiking. Figure 6b depicts a raster plot of CA1 PCs spiking. It is evident that the PCs are active 1–3 times during a recall cycle, with their spiking activity being a perfect match to the stored pattern. Eight recall half-cycles are shown, following an initialization period of 50 ms. Recall quality (RQ) is calculated by measuring the CA1 PC spiking activity during each gamma cycle (Figure 6c shows spike counts in gamma cycle). For each gamma cycle window, a binary vector of length 100 is formed, with entries having a value of 1 if the corresponding PC spikes in the window. The correlation [normalized dot product; Equation (1)] of this vector with the expected pattern vector is calculated to give a measure of recall quality between 0 and 1, with 1 corresponding to perfect recall. Figure 6d depicts RQ over time. All recall events depicted in Figure 6 are perfect, and MRQ is 1. Figure 7 shows voltage traces from a CA1 PC that belongs to the pattern, plus each of the four classes of interneurons. The phase relationships of each cell firing can be seen.

### 4.2. Effects of Spurious EC Input During Pure Recall of Previously Stored Patterns

**Figure 8:**
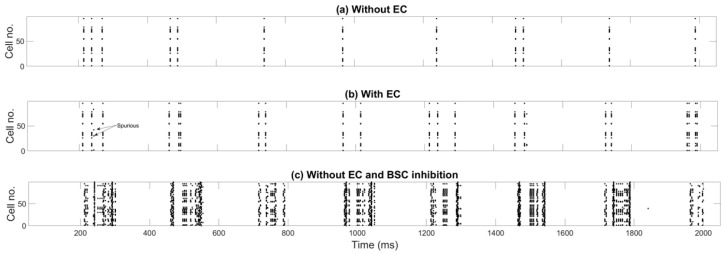
Raster plots of EngC activity during a recall episode similar to that of Figure 6. (**a**) Same network configuration as in Figure 6, with EC input disconnected from PCs. (**b**) EC input is now connected to PCs, which provides some recall cueing. (**c**) EC input is disconnected from PCs, and BSC inhibition in PC dendrites is completely removed.

Up to now, we have considered pure recall when EC input was disconnected from the PCs. An EC input corresponding to the cued pattern could potentially aid recall. Figure 8a,b shows PC recall activity without (Figure 8a) and with (Figure 8b) contribution from and 100% similar to the CA3 cue EC input. With EC input, the pattern is nearly perfectly recalled on each gamma cycle during a recall theta half-cycle (some spurious firing can be seen), and MRQ averages at 0.98.

**Figure 9:**
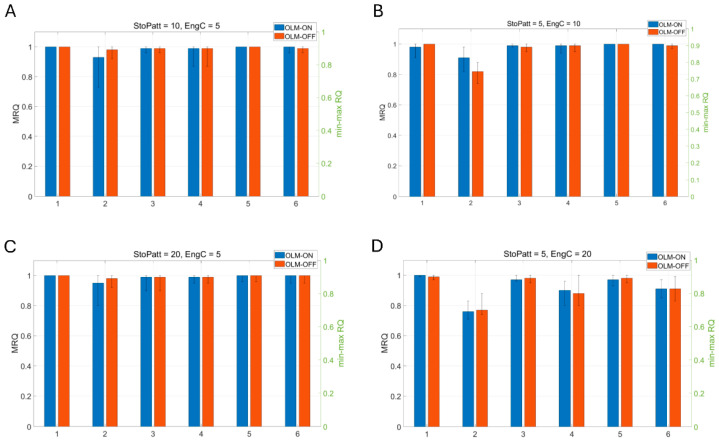
Mean recall quality (MRQ) (left y-axis) of six models when (**A**) 10 memory patterns with 40% overlap were stored and 5 engram cells coded each of these memory patterns. MRQ for each model with OLM-ON was: 1 (model 1), 0.93 (model 2), 0.99 (model 3), 0.99 (model 4), 1 (model 5), 1 (model 6). MRQ for each model with OLM-OFF was: 1 (model 1), 0.98 (model 2), 0.99 (model 3), 0.99 (model 4), 1 (model 5), 0.99 (model 6). (**B**) 5 memory patterns with 40% overlap were stored, and 10 engram cells coded each of these memory patterns. MRQ for each model with OLM-ON was: 0.98 (model 1), 0.91 (model 2), 0.99 (model 3), 0.99 (model 4), 1 (model 5), 1 (model 6). MRQ for each model with OLM-OFF was: 1 (model 1), 0.82 (model 2), 0.98 (model 3), 0.99 (model 4), 1 (model 5), 0.99 (model 6). (**C**) 20 memory patterns with 40% overlap were stored, and 5 engram cells coded each of these memory patterns. MRQ for each model with OLM-ON was: 1 (model 1), 0.95 (model 2), 0.99 (model 3), 0.99 (model 4), 1 (model 5), 1 (model 6). MRQ for each model with OLM-OFF was: 1 (model 1), 0.98 (model 2), 0.99 (model 3), 0.99 (model 4), 1 (model 5), 1 (model 6). (**D**) 5 memory patterns with 40% overlap were stored, and 20 engram cells coded each of these memory patterns. MRQ for each model with OLM-ON was: 1 (model 1), 0.76 (model 2), 0.97 (model 3), 0.9 (model 4), 0.97 (model 5), 0.91 (model 6). MRQ for each model with OLM-OFF was: 0.99 (model 1), 0.77 (model 2), 0.98 (model 3), 0.88 (model 4), 0.98 (model 5), 0.91 (model 6). Error bars indicate min–max RQ values (right y-axis). OLM-ON, OLM inhibition inhibiting the LM dendrites of all PCs in the network was active (NPC = 100); OLM-OFF, OLM inhibition inhibiting the LM dendrites of all PCs in the network was inactive (NPC = 100). EC input drove the engram cells of the CA3 cue pattern (see Figure 12A).

**Figure 10:**
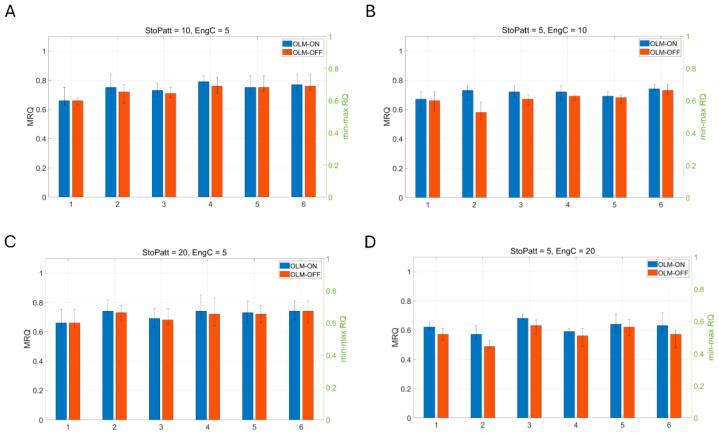
Mean recall quality (MRQ) (left y-axis) of six model variants when (**A**) 10 memory patterns with 40% overlap were stored and 5 engram cells coded each of these memory patterns. MRQ for each model with OLM-ON was: 0.66 (model 1), 0.75 (model 2), 0.73 (model 3), 0.79 (model 4), 0.75 (model 5), 0.77 (model 6). MRQ for each model with OLM-OFF was: 0.66 (model 1), 0.72 (model 2), 0.71 (model 3), 0.76 (model 4), 0.75 (model 5), 0.76 (model 6). (**B**) 5 memory patterns with 40% overlap were stored, and 10 engram cells coded each of these memory patterns. MRQ for each model with OLM-ON was: 0.67 (model 1), 0.73 (model 2), 0.72 (model 3), 0.72 (model 4), 0.69 (model 5), 0.74 (model 6). MRQ for each model with OLM-OFF was: 0.66 (model 1), 0.58 (model 2), 0.67 (model 3), 0.69 (model 4), 0.68 (model 5), 0.73 (model 6). (**C**) 20 memory patterns with 40% overlap were stored, and 5 engram cells coded each of these memory patterns. MRQ for each model with OLM-ON was: 0.66 (model 1), 0.74 (model 2), 0.69 (model 3), 0.74 (model 4), 0.73 (model 5), 0.74 (model 6). MRQ for each model with OLM-OFF was: 0.66 (model 1), 0.73 (model 2), 0.66 (model 3), 0.72 (model 4), 0.72 (model 5), 0.74 (model 6). (**D**) 5 memory patterns with 40% overlap were stored, and 20 engram cells coded each of these memory patterns. MRQ for each model with OLM-ON was: 0.62 (model 1), 0.57 (model 2), 0.68 (model 3), 0.59 (model 4), 0.64 (model 5), 0.63 (model 6). MRQ for each model with OLM-OFF was: 0.57 (model 1), 0.49 (model 2), 0.63 (model 3), 0.56 (model 4), 0.62 (model 5), 0.57 (model 6). Error bars indicate min–max RQ values (right y-axis). OLM-ON, OLM inhibition inhibiting the LM dendrites of all PCs in the network was active (NPC = 100); OLM-OFF, OLM inhibition inhibiting the LM dendrites of all PCs in the network was inactive (NPC = 100). EC input drove 40% engram cells and 60% non-engram cells of the CA3 input cue (see Figure 13A).

**Figure 11:**
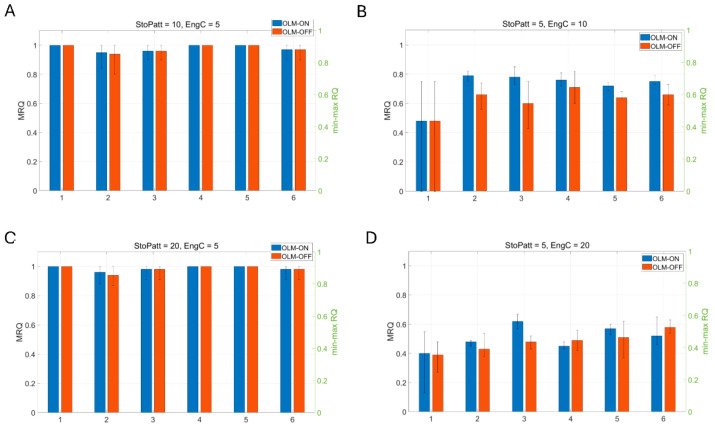
Mean recall quality (MRQ) (left y-axis) of six model variants when (**A**) 10 memory patterns with 40% overlap were stored and 5 engram cells coded each of these memory patterns. MRQ for each model with OLM-ON was: 1 (model 1), 0.95 (model 2), 0.96 (model 3), 1 (model 4), 1 (model 5), 0.97 (model 6). MRQ for each model with OLM-OFF was: 1 (model 1), 0.94 (model 2), 0.96 (model 3), 1 (model 4), 1 (model 5), 0.97 (model 6). (**B**) 5 memory patterns with 40% overlap were stored, and 10 engram cells coded each of these memory patterns. MRQ for each model with OLM-ON was: 0.48 (model 1), 0.79 (model 2), 0.78 (model 3), 0.76 (model 4), 0.72 (model 5), 0.75 (model 6). MRQ for each model with OLM-OFF was: 0.48 (model 1), 0.66 (model 2), 0.6 (model 3), 0.71 (model 4), 0.64 (model 5), 0.66 (model 6). (**C**) 20 memory patterns with 40% overlap were stored, and 5 engram cells coded each of these memory patterns. MRQ for each model with OLM-ON was: 1 (model 1), 0.96 (model 2), 0.98 (model 3), 1 (model 4), 1 (model 5), 0.98 (model 6). MRQ for each model with OLM-OFF was: 1 (model 1), 0.94 (model 2), 0.98 (model 3), 1 (model 4), 1 (model 5), 0.98 (model 6). (**D**) 5 memory patterns with 40% overlap were stored, and 20 engram cells coded each of these memory patterns. MRQ for each model with OLM-ON was: 0.4 (model 1), 0.48 (model 2), 0.62 (model 3), 0.45 (model 4), 0.57 (model 5), 0.52 (model 6). MRQ for each model with OLM-OFF was: 0.39 (model 1), 0.43 (model 2), 0.48 (model 3), 0.49 (model 4), 0.51 (model 5), 0.58 (model 6). Error bars indicate min–max RQ values (right y-axis). OLM-ON, OLM inhibition inhibiting the LM dendrites of all PCs in the network was active (NPC = 100); OLM-OFF, OLM inhibition inhibiting the LM dendrites of all PCs in the network was inactive (NPC = 100). EC input drove non-engram cells of the CA3 cue pattern (see Figure 14A).

**Figure 12:**
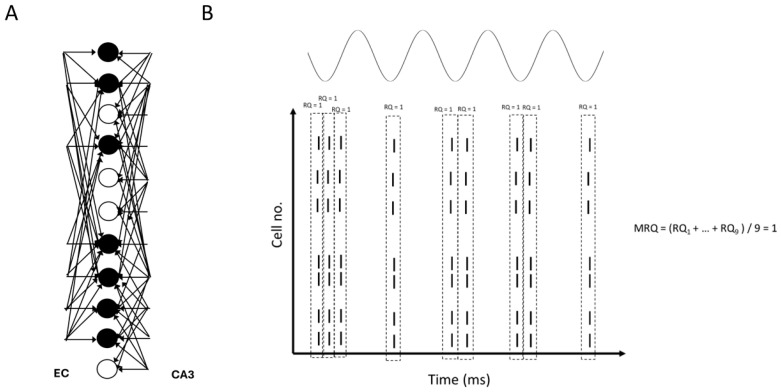
(**A**) Model inputs to the CA1 microcircuit. Solid black circles: engram cells. Hollow circles: non-engram cells (spurious cells). (**B**) Schematic of theta recall when EC drove the engram cells in the CA3 input cue. EC acted as a boosting signal to the engram cells, causing them to sometimes fire in more than one gamma cycle. RQ in each gamma cycle was 1, and MRQ averaged at 1.

**Figure 13:**
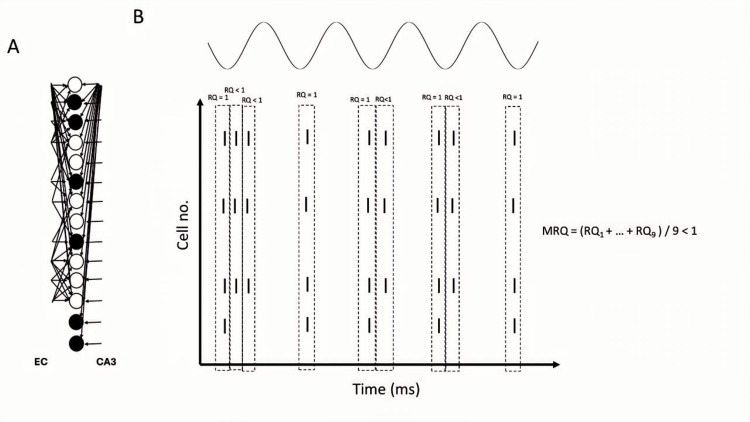

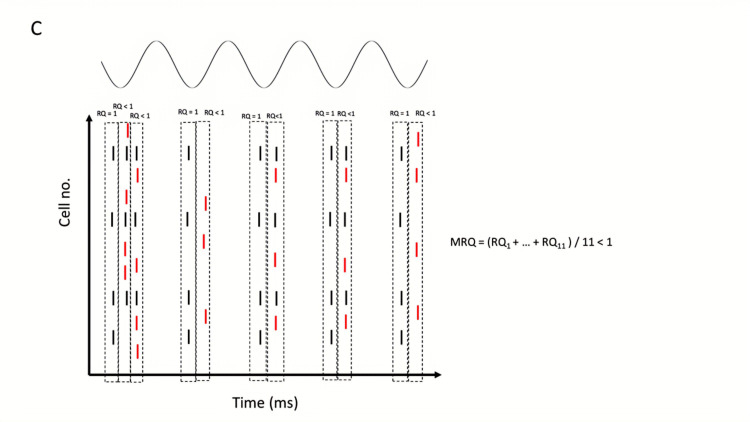
(**A**) Model inputs to the CA1 microcircuit. Solid black circles: engram cells. Hollow circles: non-engram cells (spurious cells). (**B**) Schematic of theta recall when EC drove 40% of the engram cells and 60% of non-engram cells in the CA3 input cue. Five theta recall cycles are depicted. EC acted as a boosting signal to the 40% engram cells in the CA3 cue, causing them to sometimes fire in more than one gamma cycle. OLM inhibition was ON. RQ in each first gamma cycle of each recall cycle was 1, but RQ in subsequent gamma cycles of each recall cycle was less than 1 because only 40% of engram cells fired spikes, so MRQ averaged at less than 1. (**C**) OLM inhibition was OFF. Spurious activities depicted in red are present during each 2nd or 3rd gamma cycle. MRQ averaged at less than 1.

**Figure 14:**
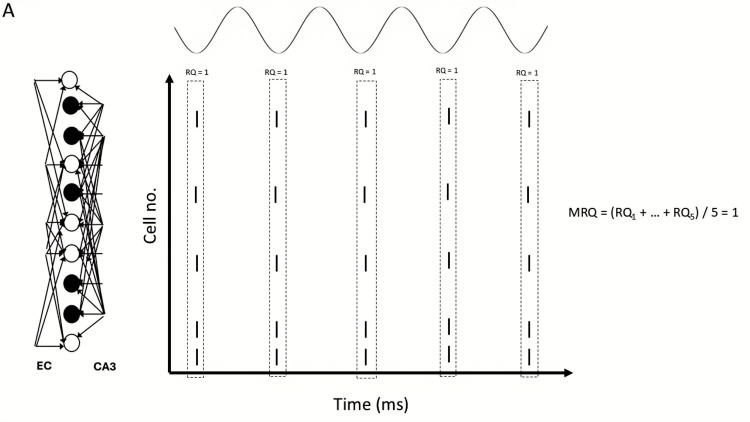

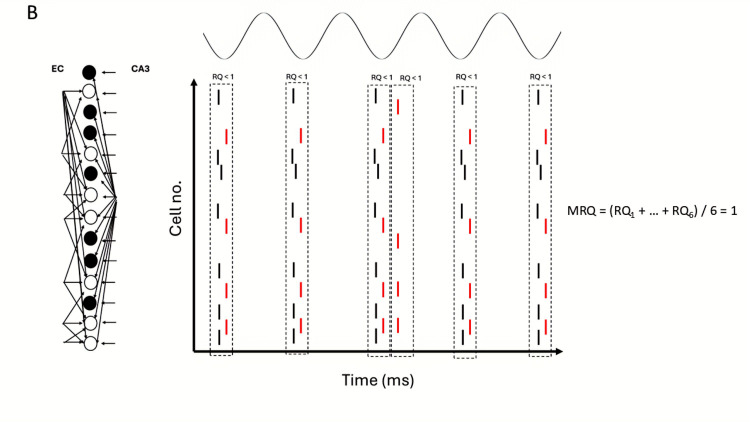
Schematic of theta recall when EC drove only the non-engram cells in the CA3 input cue. Five theta recall cycles are depicted. EC acted as a boosting signal to these non-engram cells in the CA3 cue, causing them to sometimes fire in some gamma cycles. Solid black circles: engram cells. Hollow circles: non-engram cells (spurious cells). (**A**) Population of non-engram cells was small. RQ in each gamma cycle of each recall cycle was 1, and MRQ averaged at 1. (**B**) Population of non-engram cells excited by EC was large. Spurious activities depicted in red are present during each gamma cycle. RQ in each gamma cycle of each recall cycle was less than 1, so MRQ averaged at less than 1.

To test the influence of the inhibitory pathways on recall, different pathways are selectively removed. Complete BSC inhibition removal from the network leads to firing of virtually all PCs during the recall cycles (see Figure 8c). MRQ averages at 0.25. Next, we tested complete removal of OLM inhibition when a spurious EC input was connected to the CA1 PCs’ apical dendritic tufts throughout the theta cycle: (*Case 1*) EC drove only the engram cells of the CA3 input cue and none of the non-engram ones (see Figure 12A), (*Case 2*) EC drove 40% of engram cells and 60% of non-engram cells of the CA3 input cue (see Figure 13A), and (*Case 3*) EC drove only the non-engram cells of the CA3 input cue and none of the engram ones (see Figure 14A-left). Mean recall quality (MRQ) in this situation, with and without OLM inhibition to the distal apical PC dendrites, is shown in Figure 9, Figure 10 and Figure 11. Recall quality of a pattern was calculated as described in the previous section. MRQ was calculated by adding the RQ of each pattern from a set of stored patterns and dividing this sum by the total number of stored patterns (see Equation (2)). In Case 1, recall was almost perfect in all conditions (EngC, StoPatt, and Models) with/without OLM inhibition (see Figure 9). Recall was disrupted in Case 2, and this disruption was worse when the OLM inhibition was absent (OLM-OFF) (see Figure 10). When EC drove only the non-engram cells (Case 3), then recall was disrupted only when the population of cells that belonged to the pattern (engram cells) was increased (EngC = 10 or EngC = 20) (see Figure 11). The disruption was once again worse when the OLM inhibition was absent (OLM-OFF) (see Figure 11). Interestingly, when the population of engram cells was small (EngC = 5) and EC and CA3 drove different PCs, then recall was almost perfect in all models (see Figure 11). Removal of OLM inhibition in this case disrupted recall only when the population of engram cells was large (see Figure 11).

## 5. Discussion

### 5.1. Model Predictions

A detailed biophysical model of the rat hippocampal CA1 microcircuit [36] was employed to computationally investigate the role of dendritic inhibition in memory retrieval during a multiplicative interaction of SC and PP inputs. The model successfully simulated the timing of firing of different hippocampal cell types relative to the theta rhythm in anesthetized rats [28,30,31]. The model proposed functional roles for two types of dendritic inhibition in the retrieval of information.

Specifically, our study showed that BSC inhibition in the apical PC dendrites mediated thresholding of PC firing during memory retrieval by restricting the depolarizing signals in the dendrites of non-engram cells, thus preventing them from firing, and ensuring perfect memory retrieval (only engram cells fire). In contrast, OLM inhibition in the apical dendritic tuft removed interference from spurious EC during recall. Of the three cases tested, when EC drove the engram cells of the CA3 input cue (*case 1*), then MRQ was almost perfect under all tested conditions (stored patterns, interference, engram cells, models). This was because the EC input operated as a boosting signal to the engram cells, ensuring their activities would overcome the BSC threshold inhibition and fire during memory retrieval. Engram cells were active one to three times during a recall cycle, with their spiking activity being a perfect match to the stored pattern (see Figure 12). Because EC input targeted only the engram cells and not the non-engram ones, removal of the non-specific apical tuft inhibition (OLM) to all PCs (engram and non-engram) had no effect on recall quality. However, in *Case 2*, when EC drove 40% of engram cells and 60% of non-engram cells in the cue (CA3 input) (see Figure 13A), then recall was disrupted (MRQ ranged between 0.6 and 0.8), and this disruption was worse when the apical tuft inhibition was removed. This was because the EC input operated as a boosting signal to only 40% of the engram cells, causing only those to overcome the BSC inhibition and fire 2–3 times during each recall cycle. The other 60% of engram cells fired only in the first gamma cycle due to the CA3 cue input, but not in the subsequent ones of each recall cycle (see Figure 13B), and hence RQ was reduced. Since MRQ was the average RQ from all gamma cycles, and the RQ of the 2nd or 3rd gamma cycle in each recall cycle was disrupted (not all engram cells fired in these gamma cycles), the MRQ value was reduced. Removal of the OLM apical dendritic tuft inhibition caused more spurious cells (non-engram cells) to be active during recall, which disrupted the MRQ even further (see Figure 13C). In *Case 3*, when EC drove the non-engram cells of the CA3 input cue, then recall was perfect again, but only when the population of engram cells was small (EngC =5). As the population of engram cells increased (EngC = 10, or EngC = 20), then MRQ was disrupted. Figure 14A depicts a schematic of recall when EC input drove only a small population of non-engram cells of the CA3 cue input. EC acted as a boosting signal to these non-engram cells. However, because each of these non-engram cells was activated by only 5 EC input spikes, which, even though operated as a boosting signal, this boost was not sufficient to overcome the BSC apical dendritic threshold inhibition and cause these spurious cells to fire action potentials. Hence, only engram cells fired during gamma in each recall cycle. Thus, MRQ averaged at 1. However, when EC input drove a larger population of non-engram cells of the CA3 cue input, and each of these cells received excitation by a larger number of EC spikes, then the boost was sufficient to overcome the BSC threshold inhibition and spike during a recall cycle (see Figure 14B). Spurious activities depicted in red are evident in a gamma cycle. MRQ averaged between 0.4 and 0.8 (see Figure 11B,D). Removal of the OLM apical tuft inhibition (i.e., the mechanism to remove spurious interference), disrupted recall performance even further (MRQ < 0.4 in Model 1) (see Figure 11D), particularly when the population of engram cells was large (EngC= 10 or EngC = 20) (see Figure 11B,D).

### 5.2. Model Extensions and Alternatives

Several extensions to this work deserve further consideration. The microcircuit considered in this study is still very simple compared with what we know about the biological CA1 microcircuit. More cell types and their connectivity could be included in the model. Experimental evidence has shown two more types of dendritic inhibitory cells, namely the neurogliaform (NGL) and IVY cells, which are also theta-modulated [43,44,45,46]. In our model, only the synapses on the apical dendrites of PCs are modifiable. In reality, though, most, if not all, synaptic pathways are modifiable. For example, the excitatory synapses in the apical dendritic tufts of CA1 PCs driven by the EC input are modifiable, and the postsynaptic signals in these dendrites are under inhibitory and neuromodulatory control [4,47]. Excitatory synapses on inhibitory cells and inhibitory synapses on excitatory cells have been shown to be plastic [48,49,50]. A computational study has shown that inhibitory plasticity modulates homeostasis at the network level [51]. However, very little is known about what learning rules govern these plastic synapses.

## 6. Conclusions

A neuromorphic microcircuit model of the rat hippocampus was presented in this paper, which uncovered the functional roles of dendritic inhibition impinging on apical and apical tuft dendritic arbors of CA1 PCs. Our model’s predictions offered important insights into new treatments in Alzheimer’s disease and dementia because it deciphered which synaptic pathways in the hippocampus may be (pharmacologically) treated to enhance memory retrieval and remembering.

## Data Availability

No data were used nor generated from this theoretical work, other than what is displayed in the figures. Model source code is available upon request to the corresponding authors.
